# Medical treatment of open-angle glaucoma

**Published:** 2012

**Authors:** Fatima Kyari, Mohammad Abdull, Dan Kiage, Adunola Ogunro

**Affiliations:** Ophthalmologist, Department of Ophthalmology, College of Health Sciences, University of Abuja, Nigeria.; Ophthalmology Department, Abubakar Tafawa Balewa University Teaching Hospital, Bauchi, Bauchi State, Nigeria.; Head of Ophthalmology, Aga Khan University Hospital, Kenya.; Consultant ophthalmologist and glaucoma specialist, James Standefer I Glaucoma Institute, Lagos, Nigeria.

**Figure:**
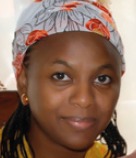
Fatima kyari

**Figure:**
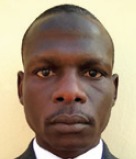
Mohammad Abdull

**Figure:**
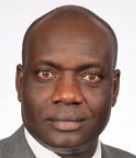
Dan Kiage

**Figure:**
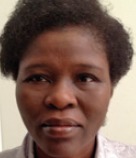
Adunola Ogunro

Most guidelines that exist for the treatment of primary open-angle glaucoma (POAG) – such the National Institute for Health and Clinical Excellence (NICE) guidelines (page 47) – recommend medicine as initial therapy, though laser treatment may also play a role.

None of these guidelines are based on research done in Africa, however, and there also is no evidence yet on the relative effectiveness of different glaucoma medications in African populations.

For medicines to be effective at controlling POAG, they must be used every day for the remainder of the patient's life.

This means the medicine must not only be clinically effective, it must also be available, of good quality, affordable, and well tolerated by the patient.

Keep the following in mind when choosing medical treatment for your patient.

1. The cost: can the patient afford the recommended medication in the long term?
2. Availability: will the drug(s) always be in stock, and what are the consequences to the patient of any stock-outs?
3. Quality: for drugs such as latanoprost, will the cold chain be maintained? Can the patient refrigerate the medicine? Can the patient recognise and avoid fake drugs?
4. Discomfort: is there any discomfort associated with the medicine that will discourage the patient from continuing with treatment?
5. Follow-up: will the patient be able to attend regular follow-up appointments?

**‘Medicines must be used every day for the rest of the patient's life’**

If you have concerns about any of the above, it may be advisable to consider surgery to control intraocular pressure (IOP). Ongoing monitoring is essential during medical treatment. The NICE guidelines suggest that surgery should be offered if two drugs (or one fixed-combination drug preparation) are not sufficient to control pressure and/or halt disease progression.

## Choosing the right drugs

There are five main groups of glaucoma drugs, each acting in a different way to reduce IOP:

- prostaglandin analogues (bimatoprost, latanoprost, and travoprost) increase uveoscleral outflow
- beta-blockers consist of two main groups: selective (betaxolol) and non-selective (timolol, levobunolol), both of which decrease aqueous production
- alpha-2 adrenergic agonists (apraclonidine, brimonidine) decrease aqueous production and increase uveoscleral outflow
- carbonic anhydrase inhibitors decrease aqueous formation and can be applied topically (brinzolamide, dorzolamide), or systemically (acetazolamide, methazolamide)
- parasympathomimetics (pilocarpine, carbachol) increases aqueous outflow through the trabecular meshwork by means of ciliary muscle contraction, and may open the drainage angle in angle-closure glaucoma by stimulating the iris sphincter muscle.

**Figure:**
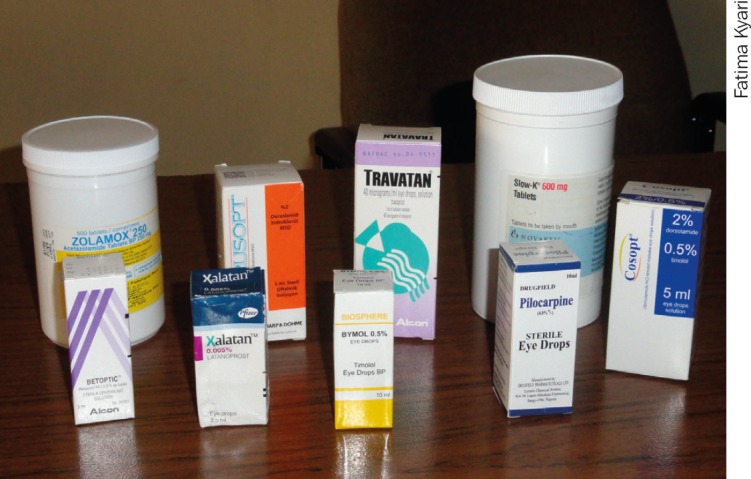
A selection of glaucoma drugs available in Nigeria

Generally, the recommended first-line drug will be one of the prostaglandin analogues (e.g., latanaprost). These drugs have an IOP lowering effect of 28–33%, require once a day dosing, and have limited local side effects. However, they are expensive and can be difficult to obtain.

Timolol, a beta-blocker, is cheaper and quite effective (an IOP lowering effect of 20–30%), but it has systemic side effects: it worsens obstructive pulmonary diseases, slows heart rate, and lowers blood pressure. Timolol 0.5% is no more effective than 0.25% is, but is much more likely to cause side effects.

If patients require more than one type of medication, use fixed combination drug preparations rather than two separate bottles. There is no evidence that fixed combinations have better outcomes than using individual drugs. However, using fixed combinations is more convenient, reduces the amount of preservatives that enter the eye, and may make it more likely that patients will continue with their treatment (known as adherence or compliance).

It is not advisable to use two or more combinations in an eye. As mentioned before, if a single combination does not work, NICE guidelines recommend offering surgery to the patient.

## Side effects

Each drug has different side effects, so prescribers and patients are advised to read inserted leaflets carefully.

- Pregnant women should avoid prostaglandin analogues (which can cause uterine contractions) and carbonic anhydrase inhibitors (which have teratogenic effects).
- People with asthma should avoid beta-blockers and parasympathomimetics (which can cause bronchiospasm).
- People with sickle-cell anaemia and/ or kidney and liver disease should avoid carbonic anhydrase inhibitors.
- People with heart block which is greater than first degree, and anyone with chronic obstructive pulmonary disease and sinus brachycardia should avoid beta-blockers.

## Useful hints

1. Determine a target IOP before starting treatment. IOP with initial single-drug therapy should be reduced by at least 20% from baseline. IOP reduction of less than 10% should be considered as a non-response.
2. The treatment goal should include stable optic nerve and nerve fibre layer status, as well as stable visual fields.
3. Switching drugs within the prostaglandin analogue class may, upon occasion, provide greater lowering of IOP.
4. Pilocarpine is useful in pigmentary glaucoma (PG) and pseudoexfoliation glaucoma (PXG), as it reduces iris movements. It may therefore reduce deposition of exfoliation material or pigment in the trabecular meshwork.
5. Topical carbonic anhydrase inhibitors (CAIs) and systemic CAIs are poorly additive with respect to lowering IOP.
6. Numerous studies have demonstrated neuroprotection in experimental models of glaucoma or optic nerve injury, but good evidence demonstrating neuroprotection in clinical studies is lacking.
7. There is insufficient evidence for neuroprotection by alpha-2 adrenergic agonists in humans.

**Patient's adherence to treatment may be encouraged and monitored by**:

- Educating and counselling the patient
- Training personnel to teach patients and their carers
- Explaining the possible side effects of each drug
- Teaching the patient to record the drugs used and instilled (page 79)
- Checking the patient's drugs at each clinic visit
- Prescribing combination drug preparations, where available, rather than many single preparations
- Giving advice to patients on how to instil eyedrops, particularly if they have any physical impairments, including visual impairment.

**How to avoid fake glaucoma drugs: top tips**

- Buy drugs from registered pharmacies
- Look out for the national drug administration/agency licensing number in your country.
- Check the manufacturing and expiry dates of drugs and be sure that these have not been altered on the packet.
- Many companies now have holograms of their logos on the packet, look for that.
- Some drug companies provide a means for patients to check the authenticity of their medicines. For example, many drug companies in Nigeria put a unique code or number on each box or bottle. Patients can SMS this free of charge to the phone number provided and the drug company will confirm whether the drug is registered and therefore genuine. This facility should be used where available
- Do not buy drugs from hawkers. Apart from raising doubts about the drug's authenticity, hawkers will not be able to store the drugs in the correct conditions. Poor storage, heat, and sunshine will decrease the potency.
- Never accept any drug package without a company label from the manufacturer (some people peel off the label and write the dosage on the bottle to hide the identity of the medication).
- If not sure of your medication, bring it to the hospital for it to be checked and confirmed.
- Be careful with expensive imported brands from big, well-known drug companies; they are more likely to be fake than locally produced drugs from smaller companies
- Ideally, eye care facilities should stock genuine drugs in good quantities and at reasonable prices. This will help ensure that patients have access to the medicines they need from a trusted source.

**Figure:**
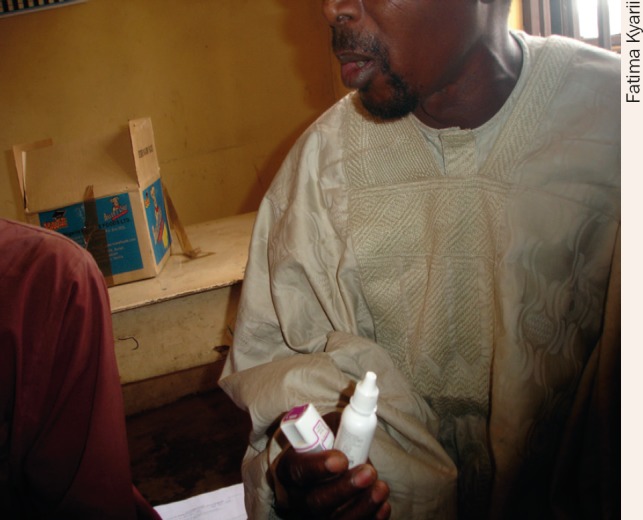
It is vital to educate patients about fake drugs and how to avoid them. Fake drugs waste money and can cost patients their sight. NIGERIA
