# Supplementary material for: Medical treatment of open-angle glaucoma
Source: Community Eye Health. 2012;25(79-80):77–9. (PMC3588128)
Supplement: Related texts

# Brief report: Instilling your own eye drops

**Published:** 2012

- If you have just been prescribed eye drops, make sure someone has shown you how to instil them. Do not leave the eye clinic until you know what to do. Make sure you have had time to practice under supervision.
- Keep this handout safe for future reference.
- Instilling your own eye drops is not easy at first, but your skill will develop as you practice. You will find a technique that works well for you but there are some points that are very important (shown in bold).
- If, after much practice, you are still struggling, ask a family member or carer to instil the eye drops for you.
- You may think it will help to use a mirror and some people may even advise this. In fact, the use of a mirror only complicates matters and can even create a dangerous technique. Avoid the use of a mirror!
- Eye drops are dispensed in various containers: a bottle (plastic or glass) with a removable dropper and combined cap; a plastic bottle with dropper attached and removable cap; or a glass bottle with plastic pipette attachment and removable cap. Whatever type is given to you, do not touch the part from which the drop falls.
- Before instilling eye drops, wash your hands thoroughly, and afterwards too.

## How to instil your own eye drops

1. Sit or lie down with your head supported.
  As your skill develops you may eventually manage to instil your eye drops while standing.
2. Use your dominant hand to hold the bottle/dropper/pipette
3. With the index finger of your other hand, hold a clean piece of tissue or cotton wool, and gently pull down the lower eyelid to form a ‘pocket’.
4. Hold the bottle/dropper/pipette between your thumb and forefinger, and place the ‘heel’ of your hand (where the wrist meets the hand) on your cheek. This will help to steady shaky hands.
5. Make sure there is a distance of about an inch (2.5 cm) between your eye and the end of the bottle/ dropper/pipette. Be careful – the tip must not touch any part of the eye or eyelids.
6. Look up or to the side. Do not look directly at the bottle/dropper/pipette.
7. Squeeze the bottle/dropper/pipette – allow one drop to fall into the lid pocket.
8. Slowly let go of the lower lid. Gently close your eyes; try not to shut them tightly as this will squeeze the drop out of your eye.
9. Dab your closed eye with the tissue or cotton wool to remove any excess.
10. Put gentle pressure on the inside corner of your eye and count to 60, very slowly. This prevents the medicine from draining out of your eye before it is absorbed.

**Figure:** Everybody develops their own technique that works for them

**A few more top tips**

**General**

- Store eye drops in a cold place, if possible in a refrigerator. It easier to feel a cold drop going in; this will reassure you that your technique is good.
- If you struggle to hold a small bottle/dropper/pipette, wrap something like a folded piece of tissue around it
- Wait at least 5 minutes between inserting different types of eye drops.
- Instil eye drops first, then eye ointment (if prescribed).

**Glaucoma patients**

- Instil your drops at regular intervals throughout the day. This is vital in controlling the intraocular pressure.
- Create a form or ‘tick sheet’ you can fill in when you have taken your drops (see Table 1).
- It is important to learn to instil your own drops. Medication for glaucoma is usually needed for the long term.
- Practice makes perfect!

**Table 1:** Sample patient record of eye drops instilled, for someone who has to instil eye drops four times a day.

Medication	Eye (left eye, right eye, both eyes?)	Breakfast/ sunrise	Lunch/midday	Evening/sunset	Bedtime
